# Case Report: Dual response to efgartigimod in myasthenia gravis and comorbid autoimmune disorders: a case series

**DOI:** 10.3389/fimmu.2026.1788550

**Published:** 2026-03-06

**Authors:** Yunjia Li, Yingying Yang, Ying Zhu, Ruixia Zhu

**Affiliations:** 1 Department of Neurology, The First Affiliated Hospital of China Medical University, Shenyang, China; 2 Key Laboratory of Neurological Disease Big Data of Liaoning Province, Shenyang, China; 3 Clinical Medical Research Center for Difficult and Serious Diseases of the Nervous Systems, Shenyang, China

**Keywords:** autoimmune encephalitis, efgartigimod, idiopathic inflammatory myopathies, IgG4-related disease, ISAACS syndrome, myasthenia gravis

## Abstract

The coexistence of myasthenia gravis (MG) with other IgG-mediated autoimmune diseases represents a clinically heterogeneous condition, driven by pathogenic autoantibodies. Efgartigimod, a neonatal Fc receptor antagonist, has the ability to clear all IgG subclasses. Here, we report four cases of MG concomitant with IgG-mediated autoimmune diseases, specifically idiopathic inflammatory myopathies (IIM), Isaacs syndrome, autoimmune encephalitis (AE), and IgG4-related disease (IgG4-RD). All of them demonstrated a favorable therapeutic response to efgartigimod, with marked improvement in QMG and MG-ADL scores, along with disease-specific clinical or laboratory manifestations of the overlapping autoimmune conditions. These cases provide preliminary evidence supporting efgartigimod as a promising treatment option for patients with MG complicated by IgG-mediated autoimmune diseases.

## 1 Introduction

Myasthenia gravis (MG) is an autoantibody-mediated neurological autoimmune disease characterized by antibodies against the postsynaptic membrane at the neuromuscular junction, leading to fluctuating skeletal muscle weakness (1). Autoimmune diseases, such as thyroid disorders, vitiligo, and SLE, co-occur in approximately 13% of MG patients. Rare neurological autoimmune disorders, such as idiopathic inflammatory myopathies (IIM), and autoimmune encephalitis (AE), have also been reported to coexist with MG (2). These conditions share common immunopathology driven by pathogenic IgG autoantibodies, HLA-linked genetic susceptibility, and thymic tolerance defects (3, 4). Previous studies have also noted associations between these IgG-mediated disorders and thymic abnormalities, thyroid autoimmunity, and thymoma-related serological markers such as anti-titin and anti-RyR antibodies, further supporting their shared mechanism (5, 6). Accordingly, efgartigimod, which reduces circulating IgG by blocking FcRn-mediated IgG recycling, may serve as a rational therapeutic strategy for MG patients with concomitant IgG-mediated autoimmune diseases (7). Here, we report four cases of MG patients who additionally present with IgG-mediated autoimmune diseases, all of whom demonstrated a favorable therapeutic response to efgartigimod.

## 2 Case presentation

### 2.1 Case 1

A 30-year-old woman was admitted to hospital in November 2024. She initially reported aching pain with swelling in the lower limbs. Days later, she developed progressive limb weakness that limited her arms elevation above shoulder level and impaired independent walking. During the week before admission, she experienced bilateral ptosis, difficulty chewing, dysphagia with choking on water, and exertional dyspnea. She underwent thymectomy for type B1 thymoma in May 2024. Upon admission, the patient was conscious and alert but exhibited dysarthria. Marked proximal weakness was present in both the upper and lower limbs. Both upper limbs were held in a flexed posture with the fingers adducted toward the palms, and extension was restricted by joint stiffness. Tenderness was noted in the gastrocnemius muscles. Laboratory testing revealed a markedly elevated creatine kinase level (1985 U/L) and positivity for anti-AChR antibody (>80 nmol/L, detected by radioimmunoassay (RIA)), anti-titin (1:100), anti-RyR (1:30), and ANA (1:320) antibodies. Myositis-specific antibodies were tested using a cell-based assay (CBA) and immunoblot, and the results were negative. Electromyography (EMG) demonstrated myopathic changes characterized by short-duration, low-amplitude, and polyphasic motor unit potentials. Repetitive nerve stimulation (RNS) revealed a significant decremental response at low-frequency stimulation. Muscle MRI displayed diffuse T2 hyperintensity involving multiple muscle groups of the right upper and lower limbs, consistent with diffuse muscle edema. Unfortunately, muscle biopsy was refused. Based on these findings, a diagnosis of anti-AChR antibody–positive generalized MG (gMG), with concurrent IIM was established (**Table 1**).

**Table 1:** Clinical characteristics of patients with myasthenia gravis and coexisting IgG-mediated autoimmune disorders treated with efgartigimod.

Case	Age/Sex	Comorbid autoimmune disease	Thymic status	Serological markers	Imaging/neurophysiology	Treatment	Outcome
1	30/F	IIM → MG (2 months)	Thymectomy (B1 thymoma)	AChR+; Titin+; RyR+; ANA+; CK ↑	Muscle MRI T2↑; EMG myopathic; RNS decrement	Efgartigimod (2 cycles) + prednisone + pyridostigmine + MMF; prednisone and MMF maintained during follow-up	QMG 28 → 2; MG-ADL 19 → 0 (MSE); CK normalized; no relapse
2	73/F	MG → Isaacs (48 months)	No thymoma	AChR+; Titin+; VGKC Ab−	EMG myokymic discharges	Efgartigimod (1 cycle) + prednisone + gabapentin + pyridostigmine; prednisone maintained during follow-up	Marked improvement in pain and stiffness; independence regained; myokymic discharges disappeared; no relapse
3	71/M	IgG4-RD → MG (48 months)	No thymoma	AChR+; IgG4 ↑	RNS decrement; CT systemic lymphadenopathy	Efgartigimod (1 cycle) + prednisone + MMF + pyridostigmine; prednisone and MMF maintained during follow-up	QMG 18 → 3; MG-ADL 16 → 2; IgG/IgG4 sustained reduction; gland enlargement resolved; no relapse
4	62/M	MG → AE (4 months)	Thymectomy (B2 thymoma)	AMPAR2 Ab +; TPOAb+; dsDNA+; AMA-M2+	Normal brain MRI; EEG generalized slowing	Efgartigimod (2 weeks) + methylprednisolone pulse followed by prednisone; MMF added during follow-up	QMG 8 → 2; MG-ADL 6 → 2; mRS 3 → 1; MMSE 24/30→27/30; no relapse

**Figure 1:**
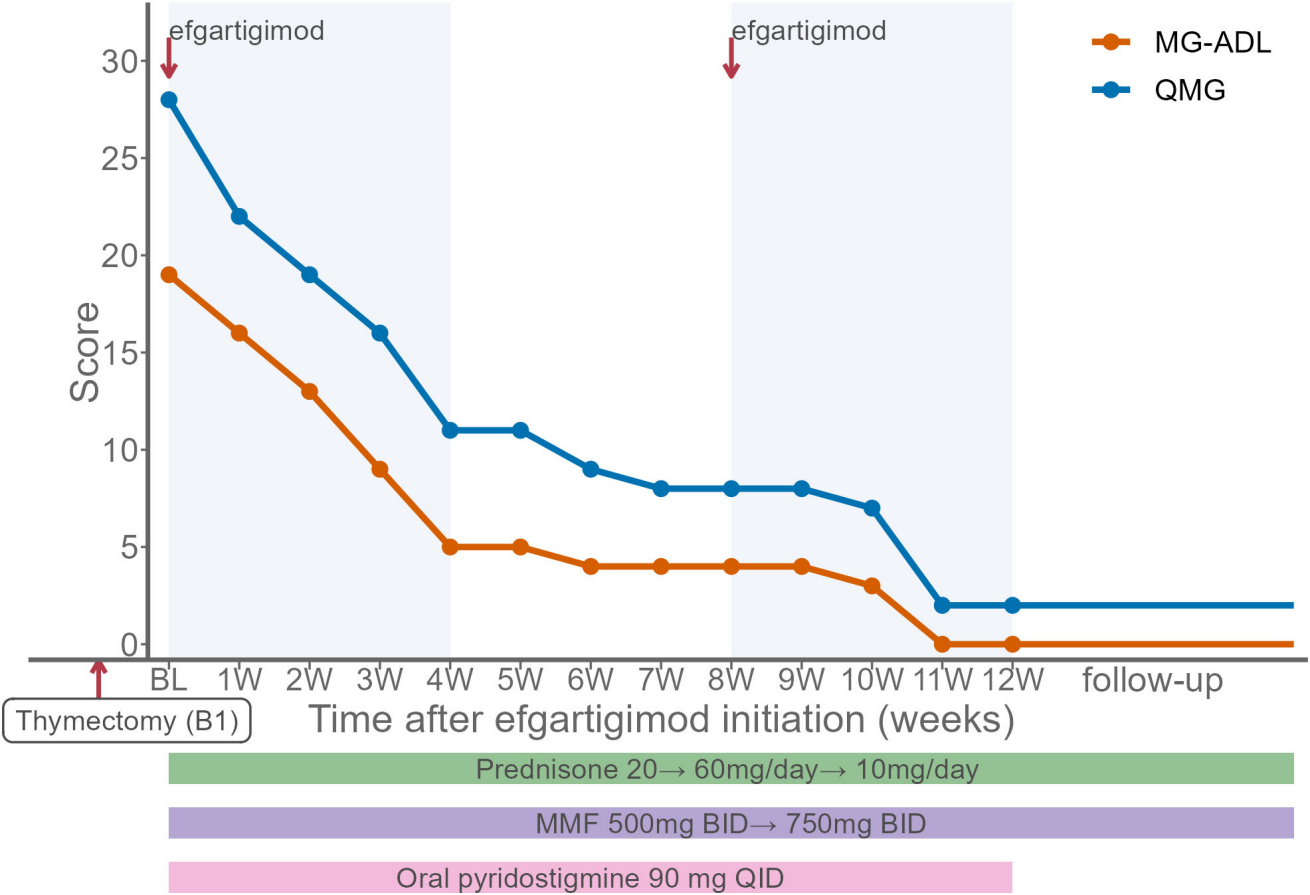
Changes in QMG and MG-ADL scores in Case 1. Baseline (BL) represents the initiation of efgartigimod. The patient had undergone thymectomy for type B1 thymoma prior to BL. Efgartigimod (10 mg/kg weekly) was administered for two 4-week cycles. At BL, concomitant therapy was initiated with oral prednisone (20 mg/day, titrated to 60 mg/day), mycophenolate mofetil (MMF, initiated at 500 mg twice daily and increased to 750 mg twice daily), and pyridostigmine (90 mg four times daily). During follow-up, prednisone was gradually tapered to 10 mg/day while MMF was maintained. Shaded areas indicate the efgartigimod treatment windows, and colored bars indicate concomitant therapies. QMG, Quantitative Myasthenia Gravis; MG-ADL, Myasthenia Gravis Activities of Daily Living.

At presentation, her Quantitative Myasthenia Gravis (QMG) and Myasthenia Gravis Activities of Daily Living (MG-ADL) scores were 28 and 19, respectively. Manual muscle testing (MMT) revealed proximal weakness, graded as 3/5 in the upper limbs, 2/5 in the lower limbs, and 3/5 in the neck flexors. Efgartigimod was administered for two 4-week cycles (10 mg/kg once weekly per cycle). Concomitantly, pyridostigmine was prescribed at 90 mg four times daily. Oral prednisone was initiated at 20 mg/day and gradually titrated by 5 mg every three days to a maximum dose of 60 mg/day. Mycophenolate mofetil (MMF) was initiated at 500 mg twice daily and titrated to 750 mg twice daily. After two weeks, marked improvement in ptosis and myalgia was observed, with QMG and MG-ADL scores decreasing to 19 and 13 (**Figure 1**). She was able to stand with assistance and swallow small amounts of water. Upon completion of the second efgartigimod cycle, her QMG and MG-ADL scores further decreased to 2 and 0, achieving minimal symptom expression (MSE). Laboratory tests also showed improvement, with serum creatine kinase decreasing to 103 U/L. Ptosis, dysphagia, dysarthria, and incomplete eyelid closure had completely resolved. With recovery of oral intake and ambulation, the nasogastric tube was removed, and she regained independent mobility. For maintenance therapy, the prednisone dose was gradually tapered from 60 mg/day to 10 mg/day, while MMF was continued. During follow-up, neurological examination demonstrated clearer speech and improved ocular and bulbar function. Muscle strength had fully recovered, with MMT improving to 5/5 in all examined muscle groups, and no relapse was observed.

### 2.2 Case 2

A 73-year-old woman received a diagnosis of MG 4 years ago, characterized by fluctuating left ptosis and diplopia. Oral pyridostigmine treatment resulted in significant symptomatic improvement. One month after the initial presentation, she developed symmetrical myalgia and cramps involving all four limbs, markedly exacerbated by exertion, particularly in the lower extremities, which progressively limited ambulation. Prednisone and azathioprine (2 tablets daily) were administered, but her symptoms persisted and impaired the daily life. In March 2025, the patient was referred to our MG clinic. Previous medical history included vitiligo and hypothyroidism, managed with levothyroxine (25 µg/day). She had left ptosis, with the eyelid covering the cornea from the 10 to 2 o’clock position. Notably, prominent and sustained muscle twitching and fasciculations were observed in both lower limbs. Strength in all four limbs was 4/5. Tone was normal and tendon reflexes were present. On serological testing, both anti–AChR antibody (>20 nmol/L, RIA) and anti-titin antibody were positive. Serum anti-voltage-gated potassium channel (VGKC) antibody was negative (CBA). EMG demonstrated myokymic discharges and fasciculation potentials occurring in doublets or triplets. Chest CT showed no evidence of thymoma. A diagnosis of simultaneous anti–AChR antibody–positive MG and Isaacs syndrome was established (**Table 1**).

One complete cycle of efgartigimod (10 mg/kg once weekly for 4 weeks) was administered in combination with prednisone (30 mg/day), pyridostigmine (60 mg three times daily), and gabapentin (300 mg three times daily). After completion of one treatment cycle of efgartigimod, she experienced marked improvement in limb pain and stiffness and regained independence in daily living. For maintenance therapy, prednisone was continued at a dose of 10 mg/day. On follow-up EMG performed approximately three months after treatment, myokymic discharges were no longer observed. No relapse has been observed during follow-up to date.

### 2.3 Case 3

A 71-year-old man presented with progressive enlargement of the lacrimal, parotid, and submandibular glands beginning in July 2024, leading to an inability to open his eyes. Xerostomia and xerophthalmia had also been present since symptom onset. Laboratory testing showed elevated total IgG (35.23 g/L), and immunoglobulin subclass testing revealed increased IgG4 (47.5 g/L). Tissue biopsy was not performed because the patient declined the procedure. The diagnosis of IgG4-related disease was made by the Department of Rheumatology and Immunology based on characteristic gland enlargement and markedly elevated serum IgG4 levels. Malignancy and other similar conditions were clinically excluded. Subsequently, intravenous methylprednisolone (40 mg/day) was administered. The gland enlargement showed partial reduction following treatment. However, new symptoms emerged, including dysphagia, dyspnea, and choking on water. Upon transfer to our department, examination showed fluctuating bilateral ptosis, diplopia, bulbar symptoms, and weakness of the masticatory and neck flexor muscles, consistent with gMG. Following a positive neostigmine test, serum anti–AChR antibody level was increased to 5.86 nmol/L (RIA). There was marked decrement on RNS testing of the right facial nerve. Chest CT showed multiple tiny pulmonary nodules with fibrotic and dependent inflammatory changes in both lungs, accompanied by enlarged mediastinal and hilar lymph nodes. Abdominal CT revealed cholelithiasis with a small gallbladder, a left renal calculus, prostatic calcifications, and multiple enlarged retroperitoneal and pelvic lymph nodes. The final diagnosis was MG concomitant with IgG4-RD (**Table 1**).

**Figure 2:**
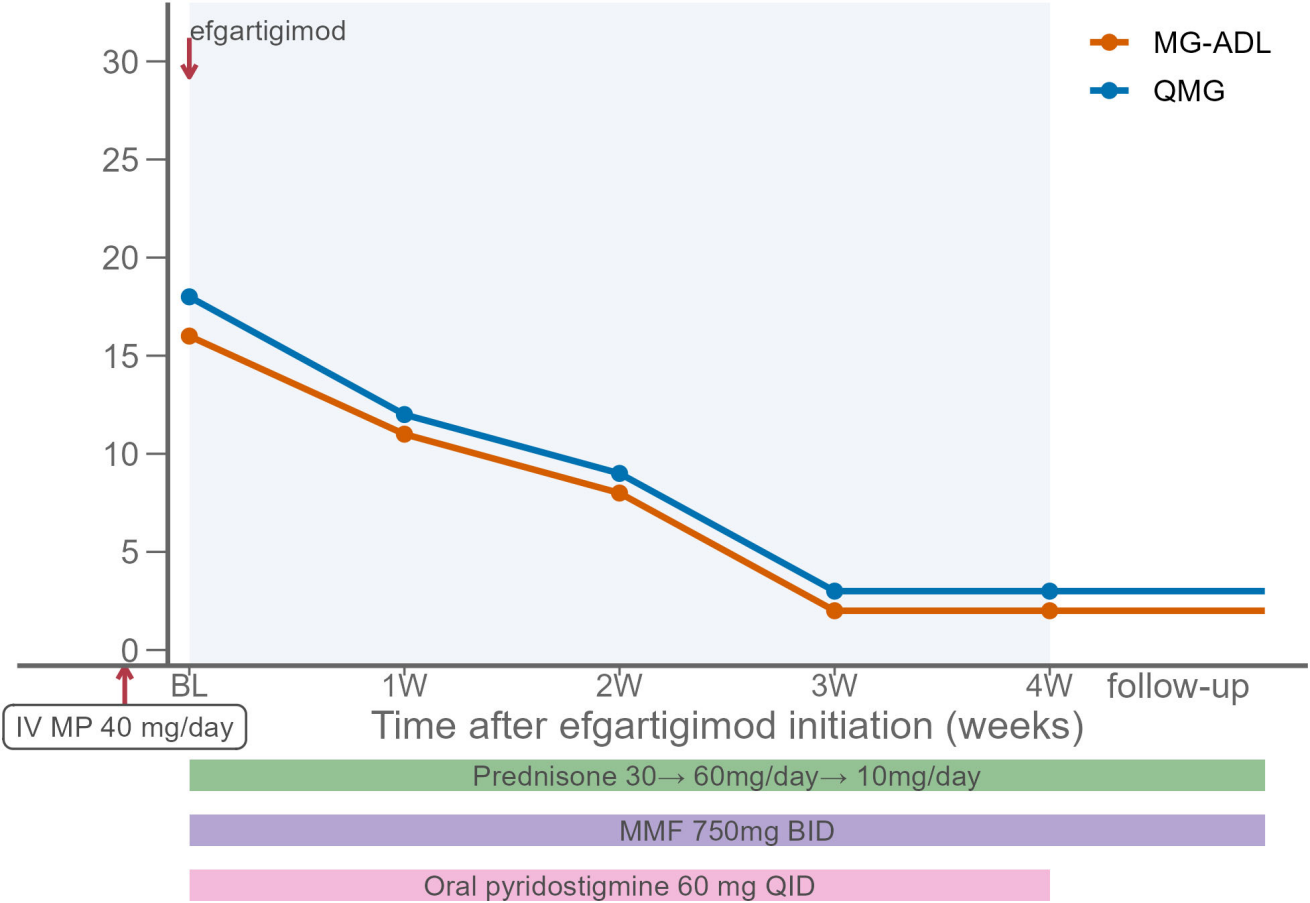
Changes in QMG and MG-ADL scores in Case 3. Baseline (BL) represents the initiation of efgartigimod (10 mg/kg weekly for 4 weeks). Prior intravenous methylprednisolone (40 mg/day, non-pulse) for IgG4-RD was ineffective. At BL, concomitant therapy was initiated with oral prednisone (30 mg/day, titrated to 60 mg/day), mycophenolate mofetil (MMF, 750 mg twice daily), and pyridostigmine (60 mg four times daily). At follow-up, prednisone was gradually tapered to 10 mg/day while MMF was maintained. Shaded area indicates the efgartigimod treatment window, and colored bars indicate concomitant therapies. QMG, Quantitative Myasthenia Gravis; MG-ADL, Myasthenia Gravis Activities of Daily Living.

**Figure 3:**
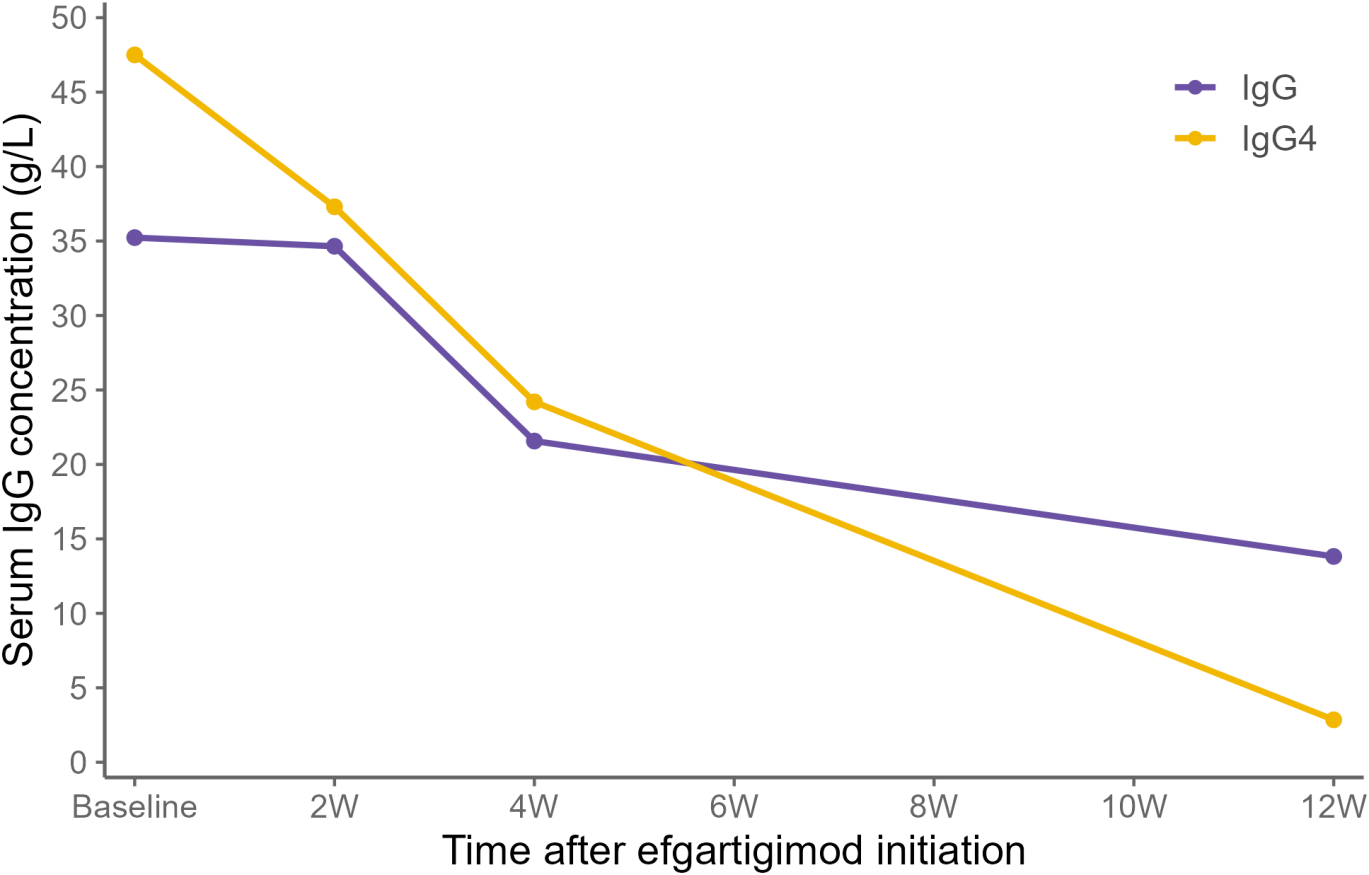
Changes in serum IgG and IgG4 levels in Case 3 over 12 weeks after initiation of efgartigimod. Baseline (BL) represents the start of efgartigimod therapy.

At baseline, his QMG and MG-ADL scores were 18 and 16, respectively. For further treatment, efgartigimod (10 mg/kg once weekly for 4 weeks) was initiated, together with MMF (750 mg twice daily) and pyridostigmine (60 mg four times daily). Prednisone was started at 30 mg/day and titrated by 5 mg every three days to a maximum dose of 60 mg/day. After four weeks, substantial clinical improvement was observed, with clearer speech and restoration of oral intake, allowing removal of the nasogastric tube. Ptosis and diplopia had improved but remained incomplete, and exertional fatigability persisted without affecting daily life, corresponding to a QMG score of 3 and an MG-ADL score of 2 at week 4 (**Figure 2**). Serum IgG and IgG4 levels showed a sustained decline over the 12 weeks following initiation of efgartigimod therapy (**Figure 3**). All previously enlarged glands regressed to normal size. He has maintained this status for 6 months, during which the prednisone dose was tapered to 10 mg/day and MMF was maintained. Regular follow-up continues in both our department and the rheumatology clinic, and no recurrence of symptoms was noted.

### 2.4 Case 4

A 62-year-old man with a history of diabetes was diagnosed with ocular myasthenia gravis (OMG) in August 2023, presenting with fluctuating left eyelid ptosis and diplopia. He responded well to oral pyridostigmine. The patient subsequently underwent thymectomy, and histopathological examination revealed a type B2 thymoma, followed by adjuvant chemotherapy and glucocorticoid 30 mg/day. In March 2024, he presented to our department with a four-month history of short-term memory impairment, preserved cued recall, and emotional irritability. Physical examination indicated that the patient was alert and oriented, with clear speech and full extraocular movements. Left eyelid ptosis was noted. Muscle strength was 4/5 in all four limbs, with normal tone and reflexes. Cognitive assessment showed mild impairment (MoCA 22/30, MMSE 24/30). The cerebrospinal fluid (CSF) examination results indicated pressure, protein, cell count, sugar and chloride measurements were normal. The anti-AMPAR2 antibody titer in serum was 1:100 and that in CSF was 1:32 (CBA). Serum anti-dsDNA, anti-PM-Scl, and anti-AMA-M2 antibodies were also detected. Cranial MRI revealed no abnormal signs. Electroencephalography showed moderate abnormalities and generalized slow activity. He was diagnosed with overlapping AE (anti-AMPAR2) and OMG after thymectomy (**Table 1**), accompanied by autoimmune thyroiditis and a possible connective tissue disease.

**Figure 4:**
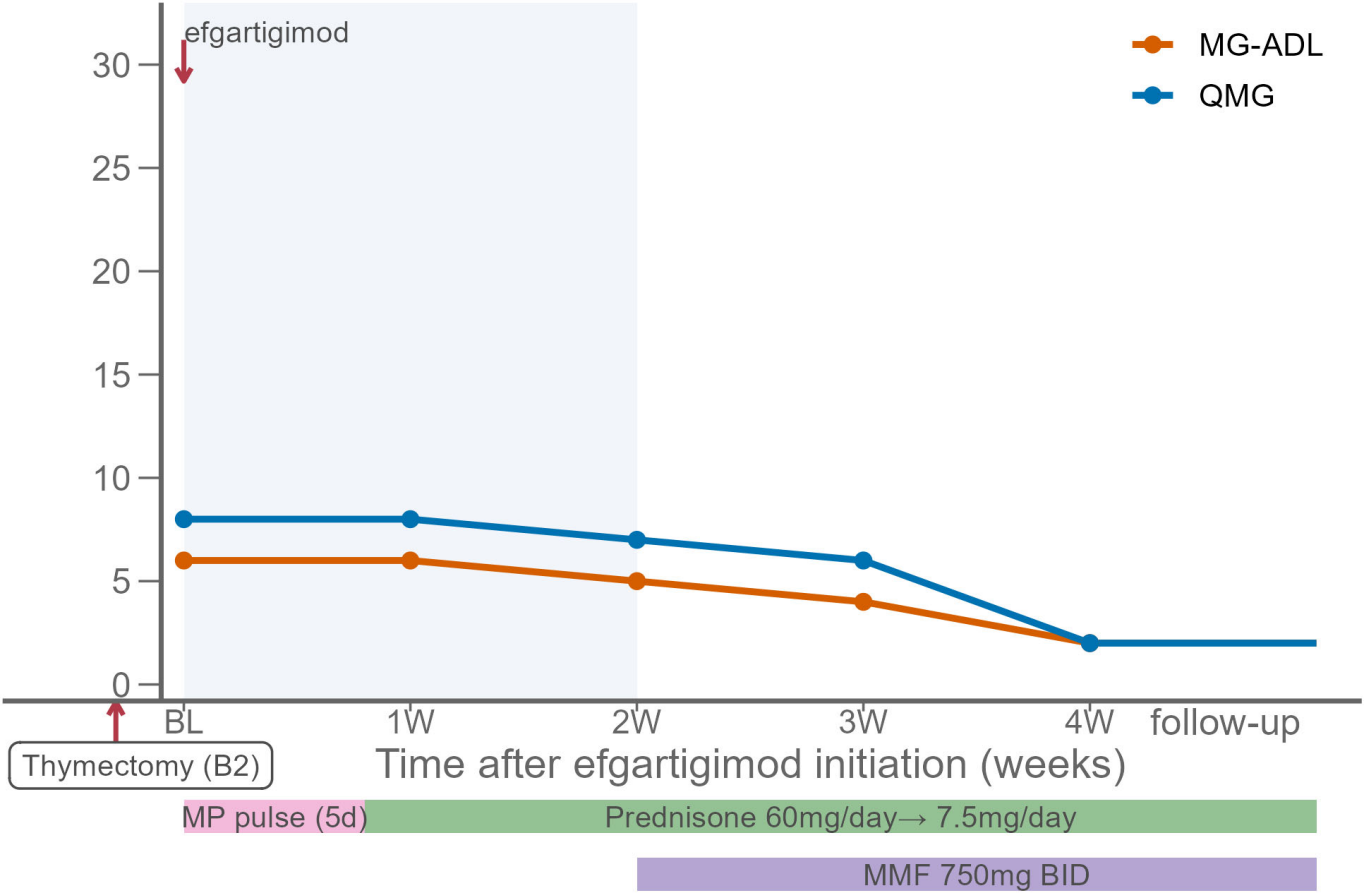
Changes in QMG and MG-ADL scores in Case 4. Baseline (BL) represents the initiation of efgartigimod (10 mg/kg weekly for 2 weeks). The patient had undergone thymectomy for type B2 thymoma prior to baseline. At baseline, a 5-day course of methylprednisolone pulse was administered, followed by oral prednisone (60 mg/day). After the 2-week efgartigimod course, prednisone was gradually tapered to 10 mg/day and mycophenolate mofetil (MMF, 750 mg twice daily) was initiated. Shaded area indicates the efgartigimod treatment window and colored bars indicate concomitant therapies. QMG, Quantitative Myasthenia Gravis; MG-ADL, Myasthenia Gravis Activities of Daily Living.

At baseline, his QMG, MG-ADL and mRS scores were 8, 6 and 3, respectively. Following a 5-day course of methylprednisolone pulse, he was transitioned to prednisone 60 mg/day. Efgartigimod (10 mg/kg once weekly) was administered for 2 weeks. Four weeks after baseline, his symptoms improved, with QMG, MG-ADL and mRS scores decreasing to 2, 2 and 1, respectively (**Figure 4**). According to family reports, his mental status became more stable compared with baseline, with improved communication and memory function. At follow-up, the MMSE score increased to 27/30 and no relapse occurred. Prednisone was gradually tapered to 7.5 mg/day, and MMF (750 mg twice daily) was added for maintenance therapy.

## 3 Discussion

In our report, four cases of MG coexisted with other IgG-mediated autoimmune diseases, specifically IIM, Isaacs syndrome, AE, and IgG4-RD. Efgartigimod resulted in significant clinical improvement in all MG patients, evidenced by reduced QMG and MG-ADL scores. Simultaneously, IgG-mediated autoimmune diseases were effectively controlled.

MG is a typical IgG-mediated autoimmune disease, characterized by pathogenic antibodies across multiple IgG subclasses, including IgG1, IgG3, and IgG4. In clinical practice, patients with MG and coexisting immune-mediated diseases are usually treated with glucocorticoids or other immunosuppressive agents, often combined with intravenous immunoglobulin or plasma exchange, with thymectomy performed in patients with thymoma. However, these treatments do not directly target pathogenic IgG, and their effects may be transient or associated with adverse effects and increased treatment burden. Recently, efgartigimod, a neonatal Fc receptor (FcRn) antagonist, has been approved for the treatment of MG and promotes sustained reduction of circulating pathogenic IgG across multiple subclasses (8). As FcRn-dependent regulation of IgG is not disease specific, this shared mechanism provides a plausible therapeutic rationale for MG patients with IgG-mediated autoimmune comorbidities.

Although MG and IIM are distinct disorders with different pathogenic mechanisms, their overlap is occasional but increasingly recognized. Wang et al. reported a 1.03% frequency of IIM-MG overlap, consistent with Uchio’s finding of 1.0% (9, 10). Compared to isolated MG or IIM, IIM-MG is characterized by dyspnea, mild CK elevation, absence of MSAs and a higher thymoma prevalence. A similar presentation was observed in patient 1. Considering the high mortality and severity, the cohort emphasized fast-acting therapies, particularly efgartigimod. IIM comprises heterogeneous subtypes (11), among which immune-mediated necrotizing myopathy (IMNM) is extensively explored with efgartigimod. Evidence from both anti-HMGCR mouse models (12) and clinical studies in refractory IMNM supports a plausible rationale for its use in our patient (13). Efgartigimod is also being evaluated in IIM in an ongoing phase II/III trial (NCT05523167). Notably, patient 1 showed marked improvement in the IIM–MG overlap syndrome following two cycles of efgartigimod therapy.

The rare overlap of MG and Isaacs syndrome has gained increasing attention due to its associated with thymoma recurrence. NMT was identified in 2% of patients in one MG cohort (14). Moreover, previous reports also described that up to 70% of NMT patients with thymoma exhibit concomitant MG with anti-AChR antibodies (15). Currently, Isaacs syndrome is increasingly recognized as an IgG4-mediated functional blockade of VGKC-complex proteins (16, 17), and IVIg has shown limited benefit in reported cases (18). Given the FcRn dependence of IgG4, efgartigimod was considered a rational therapeutic option for MG overlapping Isaacs syndrome, and the second patient improved markedly after one treatment cycle.

IgG4-RD is a systemic autoimmune disease with multiorgan involvement, and its coexistence with MG is extremely uncommon. B-cell–depleting therapy with rituximab has demonstrated efficacy in IgG4-RD and MG by suppressing pathogenic autoantibody production (19–21). Accordingly, efgartigimod was administered to patient 3, resulting in marked improvement of both MG and IgG4-RD manifestations. Notably, 12 weeks after treatment, serum total IgG and IgG4 levels declined substantially, providing further clinical evidence for efgartigimod clearance of pathogenic IgG.

Although anti-NMDAR encephalitis is the most common AE subtype, anti-AMPAR2 antibodies are reported more frequently in thymoma-associated AE and MG (22–24). Patient 4 showed a presentation consistent with published reports, including thymomatous MG preceding AE and predominant memory impairment. Immunological studies indicate that anti-AMPAR encephalitis is characterized by IgG1 anti-AMPAR2 antibodies (25). Efgartigimod has demonstrated favorable responses across NMDAR, GABABR, and LGI1 encephalitis (26, 27). Patient 4 exhibited marked improvement in both MG and AE manifestations following efgartigimod therapy, with a reduction in the mRS score.

MG and its associated autoimmune comorbidities span a heterogeneous disease spectrum. Despite their diversity across organ systems, accumulating evidence suggests that FcRn-controlled IgG homeostasis represents a shared upstream mechanism, underscoring IgG regulation and clearance as a major therapeutic focus (28). The neonatal Fc receptor, expressed on vascular endothelial cells and certain immune cells, rescues endogenous IgG antibodies from lysosomal degradation, thereby facilitating their recycling and prolonging their half-life. As an engineered human IgG1 Fc fragment, efgartigimod competitively binds to FcRn with high affinity, promoting clearance of circulating IgG. Repeated administration of efgartigimod has been reported to induce marked reductions in circulating IgG across all four subclasses, with total IgG decreases approaching 70% (8). Beyond peripheral antibody regulation, FcRn is also expressed at the blood–brain barrier, where it contributes to IgG trafficking between the systemic circulation and the central nervous system (CNS). Recent reviews further highlight efgartigimod as a therapeutic strategy across neurological autoimmune diseases, by limiting pathogenic antibodies translocation from the periphery into the CNS (7, 26). Several clinical trials are ongoing to investigate their effectiveness and safety in other neurological conditions such as neuromyelitis optica and inflammatory neuropathies (29, 30). Taken together, these findings support the therapeutic use of efgartigimod in MG with coexisting IgG-mediated autoimmune diseases. Nevertheless, this report has several limitations. The small sample size and single-center design limit the generalizability of our observations, and the observational nature of this case series limits our ability to draw causal conclusions. Further studies are needed to better understand the precise mechanisms underlying the observed therapeutic effects.

## 4 Conclusion

To date, the safety and efficacy of efgartigimod in MG have been well established. In contrast, clinical MG patients frequently present with complex autoimmune comorbidities, in which the underlying mechanisms remain elusive and therapeutic strategies are limited. To our knowledge, this is the first report describing the use of efgartigimod in patients with MG and concurrent autoimmune comorbidities. Notably, all patients achieved clinical improvement following treatment, suggesting that efgartigimod may be a promising treatment option for this regard. Moreover, subsequent clinical observations identified IMNM additional MG patients with inflammatory myopathy or LGI1-associated AE who exhibited consistent responses to efgartigimod. Larger prospective cohort studies and randomized controlled trials are warranted to further validate its therapeutic value in MG patients with concomitant IgG-mediated autoimmune disorders.

## Data Availability

The raw data supporting the conclusions of this article will be made available by the authors, without undue reservation.
